# Scoping review: quality of life of siblings of children who are deaf and hard of hearing, have a vision or motor impairment

**DOI:** 10.3389/fresc.2023.1227698

**Published:** 2023-11-14

**Authors:** Carlijn Veldhorst, Anna Luijmes, Sabina Kef, Mathijs P. J. Vervloed, Bert Steenbergen

**Affiliations:** ^1^ Behavioural Science Institute, Radboud University, Nijmegen, Netherlands; ^2^ Faculty of Social and Behavioural Sciences, University of Amsterdam, Amsterdam, Netherlands

**Keywords:** family, quality of life, disability, impairment, siblings

## Abstract

This study identified the current state of knowledge about the Quality of Life (QoL) of siblings of children who are deaf and hard of hearing (DHH), have a vision impairment (VI) or motor impairment (MI). Additionally, factors associated with individual variation in QoL were examined. A scoping review was performed using PsycInfo, ERIC, Web of Science, and CINAHL. Empirical studies with siblings (age 0-18 years) of children who are DHH, have a VI or MI that investigate the QoL of siblings were included. A total of 1746 studies were identified of which 11 met the inclusion criteria. The results showed that QoL has different interpretations and various measurement tools are used. The findings showed both positive and negative outcomes for the QoL of siblings. For example, family cohesion was found as a positive consequence. A negative consequence could be a higher score on problem behavior. Personal and parental characteristics such as age and parental availability were two main factors related to individual variation in QoL. Insight in the factors related to individual variation may help researchers to consider the research perspective. In addition, healthcare providers can use the information to be either aware or intervene on specific factors that are related to the QoL of the children who are DHH, have a VI or MI and their siblings.

## Introduction

Living with a child with a disability affects all family members. For example, previous research showed that mothers show high levels of stress (1) and low levels of self-esteem (2). In addition, siblings may face limited access to the attention of the caregiver (3, 4), higher levels of caregiving responsibility (5), and mature behavior (6) compared to their peers or act like a young carer (7–9). Although a system or family-centered approach is now very common in early intervention services for children with disabilities (10–12), these models focus mainly on the children with disabilities themselves and their caretakers, with only marginal attention paid to the siblings. These findings inspired us to review the overall quality of life (QoL) of siblings of children with physical and sensory disabilities. First, this is because, despite research emphasizing the importance of sibling support, there is still little attention for siblings in services for families with a care-intensive child with a disability (13, 14). The study of siblings, as a part of the family, is interesting in and of itself, because their wellbeing is a part of the QoL of all family members, their QoL also supports and affects the wellbeing of their sibling with a disability (15), and they cannot be omitted if rehabilitation is aimed to be family-centered (16). Second, this helps rehabilitation professionals who work in family support practices and want to include the siblings in the entire family in their services, not only clients and caretakers.

QoL is widely used in support of people with intellectual disabilities (17), which guides desired goals in rehabilitation and strategies and supports the evaluation of rehabilitation goals (18). According to the World Health Organization (WHO), QoL is a latent construct that describes subjective wellbeing and the perception of an individual of his or her position in life (19). It is a multidimensional construct (20), affected by personal and environmental factors and consisting of etic (i.e., universal) and emic (i.e., cultural) characteristics (21). QoL is operationalized in various ways, measured with different instruments tailored to the target population (22). Schalock et al. (23) conceptualized QoL as a framework comprising three factors, which were derived after structural equation modeling of data on 24 indicators and eight domains. The three factors with associated domains are independence (personal development, self-determination), social participation (interpersonal relations, social inclusion, rights), and wellbeing (emotional, physical, and material wellbeing). Although this framework is focused on people with intellectual and developmental disabilities, the framework itself is also suitable for other conditions or disabilities, as QoL consists of the same components for all people (24).

The current study has a specific focus on childhood siblings (age 0–18 years) of children who are deaf or hard of hearing (DHH), have a vision impairment (VI) or motor impairment (MI). These groups have in common that they have a sibling with a physical disability, either sensory or motor, which puts a strain on interaction and communication because of technical reasons but not so much because of a lack of cognition as in children with intellectual disabilities. A further reason to combine these three groups is that the prevalence of these disabilities is low (25).

Because of the issues and challenges that siblings of children with motor and sensor impairments have in common, it is logical to study them together. This has the additional benefit that we preserve ourselves from the diagnostic overshadowing bias (26). In diagnostic overshadowing, behaviors are assigned to the predominant disability, neglecting the fact that the same behaviors might originate for different reasons and from different causes. Diagnostic overshadowing is known to exist in the study of children with autism spectrum disorders (27) and sensory impairments (26). The term was, however, first introduced by Reiss et al. (28) to describe the tendency to assess individuals with intellectual disabilities less accurately. They made clear that diagnostic overshadowing refers to the negative bias in the judgments of the clinician if there are comorbid or co-occurring disorders in individuals who have intellectual disabilities or other mental illnesses. The symptoms that may be due to a specific mental illness are attributed to another disorder, in this case, intellectual disability, without considering alternative causes.

Siblings of children who are DHH, have a VI or MI can share experiences with each other and with siblings of children with chronic diseases or (neuro)developmental disorders, but other situations can be dissimilar (29). Although there is valuable information about siblings of children with life-threatening and chronic diseases and (neuro)developmental disorders as exemplified in recent reviews (5, 30–33), there are also important differences. Unlike chronic diseases, impairments may not compromise the health of children (19). In fact, “people with disabilities are capable of leading healthy lives” (34). Rather, children with physical or sensory disabilities may face challenges that restrict their ability to access and perform general activities, stemming from physical or social factors (35). While the existence of a disability might lead to participation problems, it is not necessarily due to bad health or life-threatening situations. Unlike somatic illnesses, disabilities may not always be treatable, which impacts hope for a cure and future expectations. This difference in experiences can also subsequently affect siblings.

Perceptions of children with disabilities have changed and now incorporate the social context (36, 37) and the overall aim to strive toward an inclusive society (38). Since general or mainstream education for children with disabilities is more common than that in previous eras, the practical implication for families is that they bear more responsibilities for education and wellbeing than previously when they shared this with clinicians and teachers (39).

The present paper examines the current state of knowledge on the QoL of siblings of children who are DHH, have a VI or MI and the factors related to individual variation. A scoping review was conducted to identify knowledge gaps and clarify key concepts (40). A scoping review was chosen because they are applicable when it is still unclear what questions can be posed (41). The review is guided by two research questions. First, what is known about the QoL of siblings of children who are DHH, have a VI or MI? Second, which factors are associated with individual variation in QoL of the siblings of children who are DHH, have a VI or MI?

## Method

### Literature search

The review was set up according to the guidelines of the Preferred Reporting Items for Systematic Review and Meta-Analyses extension for Scoping Reviews (PRISMA-ScR) checklist (42). To identify potentially relevant studies, the following databases were searched: PsycInfo, ERIC, Web of Science, and CINAHL. Note that the option “all databases” was chosen in Web of Science, which includes, among others, the Medline database. The databases were selected based on their scopes and availability. The final search strategy for PsycInfo is presented in Supplementary File S1 and was subsequently adapted for searches in all other databases. Screening for eligibility was performed in three rounds. In the first round, only abstracts and titles were screened for eligibility. In the second round, eligibility was assessed through a full-text screening. In the third round, the references of the included studies were screened for additional studies. Eligibility at both the abstract level (first round) and full-text level (second round) was assessed independently by the first and second authors or a research assistant. After that, independent assessments were compared. In the first round, both reviewers must agree to exclude a paper. Conflicting studies were included for the second round, in which any disagreements were resolved via consensus.

### Eligibility criteria

Studies were only included when they were published in English-language, peer-reviewed journals between 2002 and 2021 and studied siblings (age 0–18 years) of children who are DHH, have a VI or MI. Both qualitative and quantitative studies were included if they focused on the QoL of the siblings. We followed the conceptualization of Schalock et al. (23), as described in the Introduction section. Studies that focused on disability, disease, or neurodevelopmental disorder and that involved siblings over 18 years of age, or did not address QoL or its factors, were excluded (see also Supplementary File S2).

### Data extraction and data analysis

To answer the research questions, the first and second authors extracted the data from each included study using a data extraction list (see Supplementary File S3). For data extraction, we focused on the data relevant to our research questions. As our primary object was the QoL of siblings, we did not report results about the child with a disability or parents. The extracted data were divided and synthesized into two tables: study characteristics (see Table 1) and results (see Table 2). In addition, data were used for a narrative synthesis too. Because there was a great variety in the type of outcomes and methods of the included studies, the narrative results for the first research question were divided into outcomes, assessment tools, control groups, and positive and negative results. To describe possible factors for individual variation, the narrative results were grouped by topic.

**Table 1:** Study characteristic*s.*

#	Author	Disability or diagnosis	Participants	Age of participants	Design/method
Diagnosis leading to motor impairment					
1	Bellin et al. (44), United States of America	Spina bifida	Parents (*n *= 224), siblings (*n *= 224)	*M *= 13.81 (siblings)	Cross-sectional, questionnaire
2	Cianfaglione et al., (45), United Kingdom	Rett syndrome	Mothers (*n *= 87), siblings (*n *= 39)	*M *= 12.91 (siblings)	Cross-sectional questionnaire
3	Laufersweiler et al. (46), Germany	Spinal muscular atrophy	Children with disability (*n *= 96), siblings (*n *= 45), controls without disability (*n = *59)	*M *= 11.16 (children with disability), *M *= 11.58 (siblings), *M *= 10.50 (controls)	Cross-sectional, questionnaire
DHH					
4	Hadjikakou et al. (47), Cyprus	DHH	Parents (*n *= 30 families), siblings (*n *= 30)	*M *= 14.63 (siblings)	Cross-sectional, questionnaire, semi-structured interviews
5	Raghuraman (48), United States of America	DHH	Parents (*n *= 70), siblings (*n *= 35), controls without disability (*n* = 35)	6–12 years (siblings), 2–7 years (controls)	Cross-sectional, questionnaire, interview
6	Warner-Czyz et al. (49), United States of America	DHH	Siblings (*n *= 36)	*M *= 11.60 (siblings)	Cross-sectional, quantitative, and qualitative questionnaire
VI					
7	Haegele et al. (50), United States of America	Blindness and low vision	Parents (*n *= 22 families), children with disability (*n *= 22), siblings (*n *= 22),	*M *= 12.14 (siblings)	Cross-sectional, questionnaire
8	Hamblion et al. (51), United Kingdom	Blindness and low vision	Parents (*n *= 44), children with disability (*n *= 44), siblings (*n *= 34),	Age < 16 (siblings)	Cross-sectional, self-report, and parental proxy assessment
Mixed impairment					
9	Celik et al. (52), Poland	Neurological disability that needs physical therapy	Siblings (*n *= 50), controls without disability (*n *= 50)	*M *= 14.6 (siblings), *M *= 14.8 (controls)	Cross-sectional, questionnaire
10	Perenc et al. (53), Poland	Various, such as mobility impairment (*n *= 38), DHH (*n *= 12), VI (*n *= 8)	Siblings (*n *= 96), controls (*n *= 112)	*M *= 15.70 (siblings), *M *= 15.50 (controls)	Cross-sectional, questionnaire
11	Vella Gera et al. (54), Malta	Various, such as cerebral palsy, low vision, and deaf and hard hearing (*n *= 4)	Siblings (*N *= 7)	*M *= 10.29	Cross-sectional, qualitative, interview

DHH, deaf and hard of hearing; VI, vision impairment.

**Table 2:** Assessment tools for quality of life.

#	Goal of the study	Concept	Assessment tool for parents^a^	Assessment tool for siblings	Results
Motor impairment					
1	To investigate the relationship between individual, family, and peer factors and sibling adjustment explained by self-concept, prosocial behavior, and behavior difficulties, by using an ecological model	Psychological and behavioral adjustment	—	Feelings and attitudes about health condition [Child Attitude Toward Illness Scale (CATIS)]; satisfaction with the family interaction [Family APGAR]; perceptions of sibling warmth and conflict [Sibling Relationship Questionnaire-Brief Version (SRQ)]; perceived support of classmates and perceived support of close friends [Social Support Scale for Children (SSSC)]; global self-concept [Children's Self-Concept Scale 2 (CSCS)]; behavioral adjustment by prosocial behavior and behavior difficulties [Strengths and Difficulties Questionnaire (SDQ)]	Attitude toward illness, family satisfaction, sibling warmth and conflict, and classmate and close friend support, were strongly, positively, and significantly associated with self-concept. To a lesser degree, they were associated with prosocial behavior and behavior difficulties. Family satisfaction was the only ecological factor significantly associated with all dimensions of sibling adjustment
2	To compare the maternal mental health of mothers of children with Rett syndrome and sibling psychological adjustment to normative data and to investigate associations between maternal wellbeing and three dimensions of Rett syndrome	Psychological adjustment	Psychological adjustment of siblings (SDQ); depression and generalized anxiety in hospital settings [Hospital anxiety and depression scale (HADS)]; positive experiences raising children [Positive Gain Scale (PGS)]; impact on the parent and family [Subscale of the Questionnaire on Resources and Stress—Friedrich Short Form (QRS-F)]; extent of behavior problems of the child with Rett syndrome [Developmental Behavior Checklist (DBC)].	—	Siblings showed significantly fewer problems in psychological adjustment compared to British norms. Specifically, siblings were significantly better adjusted in the subscales of total difficulties and hyperactivity. A significantly higher proportion of the siblings scored in the abnormal range for prosocial behavior compared with British norms
3	To investigate behavioral adjustment of children with spinal muscular atrophy (SMA), their siblings, and controls	Behavior problems	Behavioral symptoms [Parental Child Behavior Checklist (CBCL)]; comorbid child psychiatric disturbances [Kinder-DIPS parent]	—	Siblings had significantly higher scores on internalizing behavior than controls. Siblings had significantly higher scores on externalizing behavior than children with a physical disability, but not significant compared to the controls
Deaf and hard of hearing					
4	To investigate psychosocial adjustment and quality of relationships of children who are deaf or hard of hearing and their siblings	Emotional and behavioral adjustment, self-perception, sibling relationship, relationship with parents, peer relationships, psychological adjustment	Siblings’ behavioral and emotional adjustment [SDQ]; sibling interactions [Sibling Inventory of Behavior (SID)]	Behavioral and emotional adjustment (SDQ); feelings about and the relationship with the child with an auditory disability [Siblings’ Problems Questionnaire (SPQ)]; self-perception [Self-Perception Profile for Children and Adolescents (SPPCA)]; sibling relationships, parent relationships, the impact of friends and children's knowledge about the disability [semi-structured interview]	The score of siblings on self-perception was rather high compared to standard mean scores and was not significantly different from standard means scores, indicating siblings were satisfied with their social, behavioral, and communication skills. Emotional and behavioral adjustment was significantly positively, associated with self-perception and global self-esteem. Siblings were worried about the future of the child with a disability and took upon themselves many responsibilities. Older siblings reported significantly more feelings of embarrassment
5	To investigate the emotional wellbeing of older siblings of children who are deaf or hard of hearing and controls	Emotional wellbeing	Behavior problems and social competence [CBCL]; child's knowledge about the disability and child's perceptions and affective responses [SPQ]; quality of relationships between siblings [SRQ]; child's activities, social interactions, and academic performance [Social Competence Scale (CBCL)]; how the child behaves and responds to different situations in everyday life [Parent Temperament Questionnaire (PTQ)]; household and child care tasks, rights and privileges, and social activities [Home Routines Assessment]	Knowledge about the child's disability, perceptions, and affective responses [Siblings Perception Questionnaire (SPQ)]; quality of relationships between siblings (SRQ)	No significant differences were found for psychological variables, parental attention, and household responsibilities. A significant, negative relation was found between the severity of hearing loss and behavioral problems. Furthermore, siblings with a more positive temperament showed significantly fewer behavior problems. Significant positive relations were found between perceiving negative relationships between siblings and interpersonal problems, and between expressing warmth/closeness and perceiving positive relationships between siblings
6	To examine the effect of having a brother or sister with a cochlear implant on the sibling with typical hearing	Perceptions, effect on sibling's activities, emotions, parental attention, and family dynamics	—	Effect on siblings (self-administrated survey with an opportunity to provide personal narratives)	The majority of the siblings reported that having a sibling with a cochlear implant does not affect them much. There was a significant relationship between the effect on the sibling and the speech intelligibility of the child with a cochlear implant. All siblings experienced a good relationship, but the extent of parental attention varied
VI					
7	To compare physical activity, nutritional intake, and psychological wellbeing of children with VI and their siblings	Psychological wellbeing	Psychological wellbeing (SDQ)	—	Siblings had significantly higher scores on prosocial behavior than their siblings with vision disabilities. No significant differences were found in the difficulties
8	To investigate the impact of the retinal disorder on the quality of life of affected children and their families	Health-related quality of life	Physical, emotional, social, and school functioning [Paediatric Quality of Life Inventory (PedsQL)]	Physical, emotional, social, and school functioning (PedsQL)	Both parents and the siblings themselves reported that the latter score significantly higher on health-related quality of life than the children with the retinal disorder and the parental score for these children. Overall, the disorder had an adverse impact on the family and family functioning
Mixed impairment					
9	To compare physical fitness, physical activity, physical state, and quality of life of siblings of children with and without a disability	Psychosocial status, quality of life	—	Psychosocial status [depression scale (CES-DC)]; quality of life [Child Health Questionnaire (CHQ)]	Siblings of a child with a disability had a significantly higher score on depression than siblings of children without a disability. No statistical differences were found for quality of life, except for the scale of family cohesion. This score was higher in the sibling group
10	To examine possible prosocial tendencies of siblings of children with disabilities	Prosocial competences	—	Prosocial tendencies [Prosocial Tendencies Scale (PTM)]	Siblings of a child with a disability reported significantly a higher overall level of prosocial tendencies compared to peers. Siblings had a significantly higher score on the subscales: anonymous, emotional, and altruism. Overall, older participants scored significantly lower in public prosocial tendencies and higher on compliance and anonymity. Boys showed a significantly higher score in public prosocial behaviors, than girls on altruism and emotions
11	To investigate the experiences of siblings of children with a disability	Experiences	—	Experiences of siblings about the themes of life as a child, connections, and perspective (semi-structured individual interview and focus-group discussion)]	Siblings had both positive and negative emotions about their sibling with a disability. Siblings indicated responsibility, maturity, independence, and a close relationship with their sibling with a disability

VI, vision impairment.

^a^Only assessment tools used to measure domains of QoL of children and parents are described.

## Results

### Selection of sources

**Figure 1:**
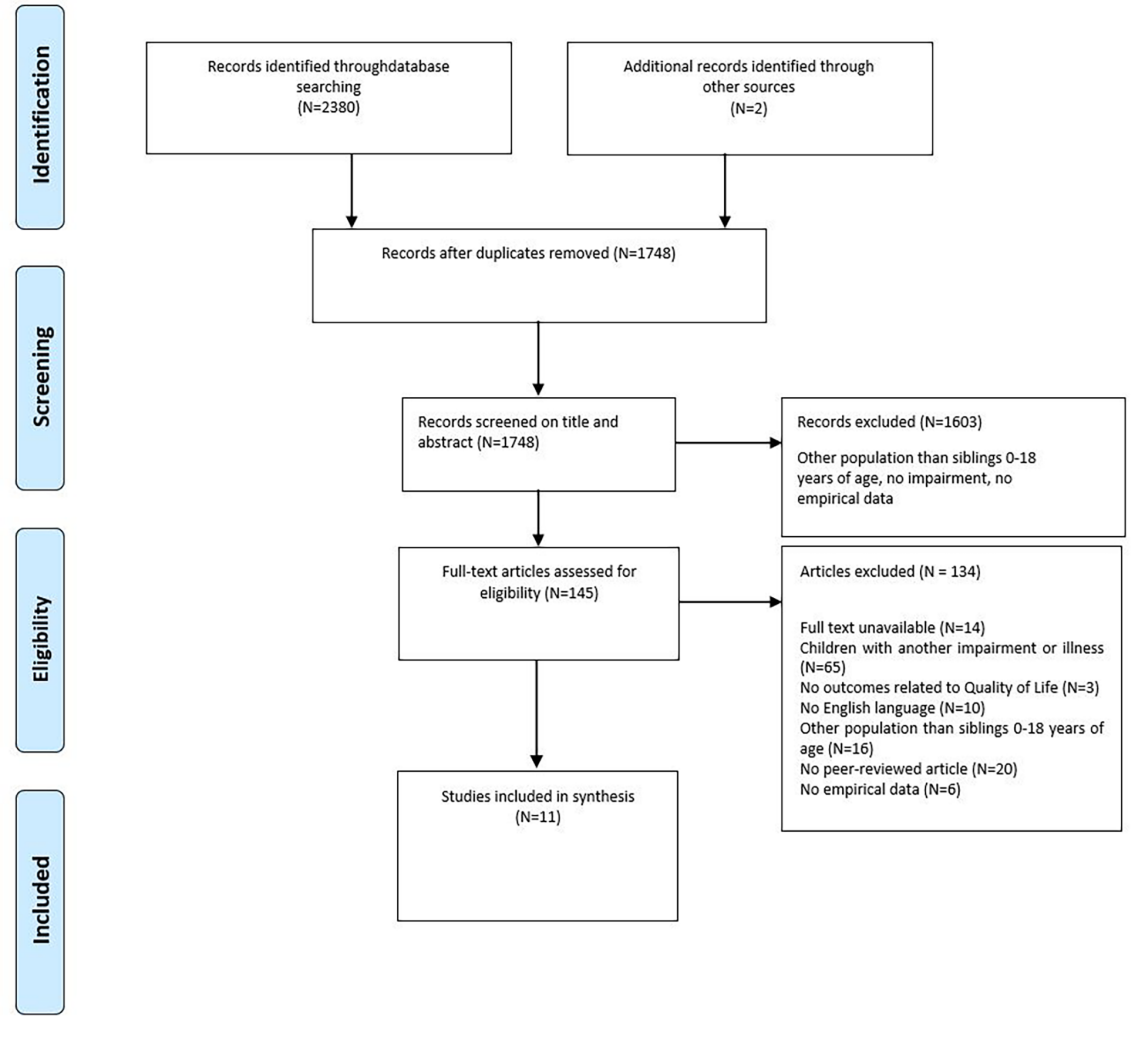
Flowchart of the inclusion process (43).

A flowchart of the selection process is depicted in Figure 1. After duplicates were removed, a total of 1,748 studies were identified. After reading titles and abstracts, 1,603 studies were excluded. Of the remaining 145 studies, no full texts could be found for the 14 studies. The other 131 studies were assessed for eligibility by full-text reading. Of the remaining studies, 65 studies were excluded because they focused on siblings of children with other conditions than being DHH, having a VI or MI or siblings older than 18 years of age (*n *= 16). In three studies, the outcomes were not related to the QoL of siblings, or the study was not empirical (*n *= 6). The manuscripts of 20 studies were not peer-reviewed, and 10 studies were not written in English. Eventually, nine studies were included for data extraction. Additional studies were searched by checking the list of references of the nine selected articles. Based on the title, 17 studies were found eligible for inclusion. These 17 studies were checked by the first and second authors in the same way as described earlier. We therefore included two of these articles that met all the inclusion criteria and yielded a final sample of 11 studies. See the flowchart in Figure 1 for an overview.

### Characteristics of sources

All studies were published between 2003 and 2021. The study characteristics are described in Table 1. Ten studies used a questionnaire to collect data (#1, 2, 3, 4, 5, 6 7, 8, 9, 10). Four studies used interviews (#4, 5, 6, 11). It differed whether parents or siblings completed the questionnaire. In six studies, parents filled in questionnaires about the siblings of children who are DHH, have a VI or MI (#2, 3, 4, 5, 7, 8). In eight studies, the siblings themselves filled in the questionnaires (#1, 4, 5, 6, 8, 9, 10, 11). In three studies, both parents and siblings completed the questionnaires (#4, 5, 8). Four studies used a control group. The controls were siblings of children without a disability (#5, 9) and typically developing children (#3, 10). Lastly, in three studies, the children with the impairment filled in the questionnaire (#3, 7, 8).

The type of impairment varied across the included studies. The studies are categorized by DHH, VIs, MIs, and mixed disabilities. With this last category, we meant all the studies that did not focus on one type of disability but had a sample of siblings of children with different types of disabilities. In Table 1, the exact type of disability can be found. The diagnoses that lead to MIs, e.g., spina bifida, Rett syndrome, and spinal muscular atrophy, were all represented once in a study. Three studies included siblings of children who are DHH, and two studies included siblings of children with VI. Finally, three studies included siblings of children with different types of disability; see Table 1. In two studies the type of impairment was degenerative (#2, 3). It is not known to what extent the impairments were progressive and degenerative. The youngest participants were 2 years of age. Two studies did not mention the mean age (#5, 8). The mean age of the siblings in the remaining nine studies was 11.36 years. The participants were recruited in several ways. Six studies used a clinical sample (#2, 3, 4, 6, 7, 8), and three studies used a community sample (#1, 5, 10). One study recruited participants via a parent association (#11). Only one study did not mention the recruitment process (#9). The recruitment resulted in a sample size of siblings between 7 and 224 across the 11 studies. The mean sample size for siblings was 53. The sample size of the parents of the siblings varied between 22 and 224 fathers and/or mothers for the 11 studies. The mean sample size for parents was 79.

### Research question 1: QoL of siblings

The first research question was about what is known about the QoL of siblings of children who are DHH, have a VI or MI. Subsequently, outcomes, assessment tools, control groups, and positive and negative consequences for the QoL of the siblings are described. See Table 2 for an overview.

#### Outcomes

The outcomes of the included studies varied widely. For clarity, we clustered studies with corresponding outcome measures, resulting in six themes, namely, QoL (overall), wellbeing, relationships, adjustment, mature behavior, and self-perception. These themes were composed by the first and second authors, based on the results of the included studies, and found their basis in the factors and domains within the QoL framework as described by Schalock et al. (23).

QoL (overall): Two studies reported results specifically about the overall QoL. No statistical differences for QoL were found between siblings of children with mixed impairments and control groups (#9), which means that there were no differences in the subscales behavior, self-esteem, and mental health. Siblings of children with VI had a higher health-related QoL compared to the child with VI (#8).

Wellbeing: The results concerning the wellbeing of siblings were mixed. No statistical differences were found between siblings of children who are DHH and controls on emotional wellbeing (#4), which indicated that the psychosocial adjustment, social competence, and self-worth of the siblings are within the normal range for their age. In contrast, siblings of children with mixed impairments reported both negative emotions, such as worries in a semi-structured interview. In addition, one study even reported a significantly higher score on depression for siblings of children with mixed impairments compared to controls (#9) and positive emotions, such as more appreciation for life (#11).

Relationships: Several studies found positive findings for relationships. Namely, siblings of children with mixed impairments had a significantly higher score on family cohesion than that of the controls (#9). In addition, in interviews, siblings had positive comments about the relationship with the child with the impairment (#6, 11), for example, about the close relationship between siblings. Nonetheless, one study, which involved families of children with VI, found an overall negative impact on family interaction (#8). In interviews, more than half of the siblings of children with mixed impairments gave negative comments about parental attention (#6), for example, that parents spend more time with the child with the impairment compared to the sibling without a disability.

Adjustment: Adjustment of siblings was reported in several studies. Siblings were found to have fewer problems in psychological adjustment compared to norm groups. More specifically they showed better adjustment on the subscales “total difficulties” and “hyperactivity” of the Strengths and Difficulties Questionnaire (SDQ) (#2). With regard to prosocial behavior, siblings showed more prosocial behavior compared to their siblings with VI (#7) and peers without a brother or sister with disabilities (#10). Furthermore, they were more likely to score in the abnormal range of the SDQ, meaning they showed high levels of prosocial behavior (#2). However, siblings showed also higher scores on internalizing behavior compared to those of control participants and higher scores on externalizing behavior compared to those of their siblings with MI (#3). However, no statistical differences were found for difficulties compared to their sibling with VI (#7).

Mature behavior: One study reported that siblings of children who are DHH had worries about the future (#4). Siblings of children who are DHH or have mixed impairments indicated in an interview to have feelings of responsibility for their sibling with a disability (#4, 11). However, no statistical differences for responsibility were found compared to siblings of children who are DHH (#5).

Self-perception: The one study that investigated self-perception found that scores on self-perception were rather high for siblings of children who are DHH, but they were not significantly different from the mean standard scores of the Greek population (#4).

#### Assessment tools

Since the outcomes of the included studies varied largely, possible variation as a result of differences in assessment tools was examined. A total of 21 different assessment tools were used in the 11 studies (see also Table 2). Only two studies used the same tool for the same indicator, namely, the SDQ to measure behavioral adjustment (#1, 4). Both studies found that behavioral adjustment had a significant positive relation with self-perception, self-esteem (#4), and self-concept (#1), respectively. In the other studies, some assessment tools were used more than once, but different aspects of the tools were used in each of these studies to describe different domains in each of these studies. Four assessment tools were used in two or more studies, namely, Parental Child Behavior Checklist (CBCL), Sibling Relationship Questionnaire (SRQ), Siblings’ Problems Questionnaire (SPQ), and the SDQ, with the SDQ being used four times (#1, 2, 4, 7). Looking at the results of these specific assessment tools, there is some consistency. Both studies #4 and #5 used the SPQ and concluded that siblings had worries about their sibling with a disability and felt very responsible for the brother or sister with a disability. Two studies used the SRQ (#1, 5), and they both concluded that there was a positive relationship between the scale warmth/closeness and other positively formulated factors, such as sibling relationship and self-concept. However, comparison groups were missing in these studies. The SDQ was used in four studies (#1, 2, 4, 7). Study #7 found that siblings had a higher score on prosocial behavior than children with VI. Both studies #2 and #4 found no significant differences compared with the norm scores. Study #1 did not compare the results of the SDQ between groups but found significant relations with family satisfaction, sibling warmth and conflict, support, and self-concept. The CBCL was used two times. The results were contradictory. In study #3, the siblings of children with MI showed significantly higher scores on problem behavior in comparison to controls. However, no significant differences were found for siblings of children with a sensory impairment (DHH or VI) in study #5.

#### Control group

hree studies did not use control groups (#1, 6, 11), whereas the remaining studies used three kinds of control groups, namely, the norm groups from the assessment tool (#2, 4), a control group consisting of siblings with the impairment (#3, 7, 8), and a control group of children who did not have a brother or sister with a disability (#3, 5, 9, 10). The latter seems to be the more appropriate control group, although the controls in those four studies were matched on only age range, not age and sex. Studies # 3, 5, 9, and 10 compared siblings of children with a disability with a control group consisting of siblings of children without a disability. It appeared that siblings of children with mixed disabilities had a significantly higher score on depression than the controls (#9). QoL was mostly equal for both groups, and only siblings of children with mixed disabilities had a significantly higher score on family cohesion. Study #3 found that siblings of children with MI scored significantly higher on internalizing behavior than the controls. Study #5 found no significant differences between the siblings and controls. In contrast, study #10 found a significantly higher overall score for prosocial tendencies for the siblings.

Studies #3, 7, and 8 compared siblings with a child with a disability. Siblings of children with VI showed significantly more prosocial behavior (#7) and had significantly higher scores on QoL (#8) than the children with VI. In contrast, siblings of children with MI showed significantly more externalizing behavior compared to their brother or sister with MI (#3).

Study #2 compared the psychosocial functioning of siblings with the norms of the assessment tool. It appeared that siblings of children with MI had significantly fewer problems related to psychological adjustment compared to norms for British children. The scores on self-perception of siblings of children who are DHH were not significantly different from the standard mean scores (#4).

#### Negative and positive consequences

The review showed that both positive and negative consequences were found for siblings. To start with the negative consequences, two studies reported siblings having more behavior problems than controls. Specifically, siblings had significantly more internalizing behavior problems than the controls and showed significantly more externalizing behaviors than the child with MI (#3). A negative correlation was found between the severity of hearing loss and behavioral problems in siblings (#5), which meant that the more severe the hearing loss the fewer behavioral problems were found in the siblings. The same study also showed that when siblings perceived their relationship with the child who is DHH to be negative, more interpersonal problems appeared (#5). Siblings of a child with a disability had a significantly higher score on depression than siblings of children without a disability (#9). Further, siblings had worries about the child with a disability and took upon themselves many responsibilities, such as helping parents with household work or taking care of their siblings (#4, 11). The older siblings were, the more they reported feelings of embarrassment (#4). Lastly, study #8 reported an impaired QoL for all family members. Overall, there was an adverse impact of the impairment on the family and family functioning. With regard to parental attention, siblings indicated that they received less parental attention compared to their siblings with a disability (#6).

At the same time, some of the reviewed studies also found several neutral and positive consequences with regard to the QoL of the siblings. The self-perception scores of siblings were not significantly different from standard mean scores (#4). The family interaction was perceived as positive (#9, 11), and no significant differences were found for emotional and behavioral adjustment, self-perception, parental attention, household responsibilities (#5), and QoL (#9). Actually, in contrast to study #8, the family scores of the latter domain were significantly higher for siblings (#9). Two studies found that siblings experienced a good relationship with their sibling with a disability (#6, 11). In addition, siblings showed more prosocial behavior (#7, 10) and better psychological adjustment compared to British norms (#2). Whether this also applies to health-related QoL is unclear, but compared to their sibling with a disability, they scored higher on health-related QoL (#8).

Next to the positive consequences for QoL, some studies also found positive associations between different domains of QoL. The attitudes of siblings toward illness, satisfaction with the family, sibling warmth and conflict, and classmate and close friend support were strongly, positively, and significantly associated with self-concept (#1). Specifically, satisfaction with the family was significantly associated with all dimensions of sibling adjustment (#1). In study #4, adjustment (emotional and behavioral) was also significantly and positively associated with self-perception and global self-esteem. Siblings who perceived positive relationships with their sibling with a disability also expressed more warmth and closeness (#5).

### Research question 2: factors associated with individual variation

The factors that are associated with individual variation in QoL of siblings of children with a disability as described in the 11 studies are listed below. From the included studies, empirical evidence for individual variation was not found for many variables. However, all studies named potential variables responsible for individual variation when discussing the results. Factors for individual variation are therefore divided into examined factors and potential factors.

#### Characteristics of the impairment

The type of impairment in relation to the outcomes on QoL of the siblings was studied. Disabilities were categorized into: DHH, VI, and MI. Studies #9, 10, and 11 were not included in this analysis because of the large heterogeneity in disabilities of the siblings of the participants. Overall, we found no general trend when analyzing the results based on the type of impairment. The complications in studying this question were the large variety of themes under study, different types of assessment tools, and the small number of studies that had comparable populations as the participants.

Impairment characteristics might be relevant for individual variation. The included studies suggested in their discussion that treatment and prognosis of the impairment can relate to individual variation in the QoL of siblings (#8) and that onset and consequences of the impairment (#1) and progressiveness (#3) may negatively impact the QoL of siblings. The impact of the severity of the impairment differed by disability group and the domain of functioning. In study #5, a significant negative association was found between the severity of hearing loss and behavioral problems of the sibling. But for siblings of children with VI in study #8, the opposite association was found for family functioning; families with a child with a severe VI reported worse family functioning. In study #1, no significant association between the severity of the MI and the QoL of siblings was found. In study #4, the possibility of communicating orally by children who are DHH had a positive impact on QoL. In addition, in study #6, siblings indicated that they felt less impacted when their sibling who is DHH had good speech intelligibility.

#### Age

The age of both the child with the disability and the sibling is associated with variation in parenting and psychosocial wellbeing. The parents indicated that older siblings of children with hearing impairments showed fewer interpersonal concerns and less fear and conflict (#5) than younger siblings. Studies #4 and 5 suggested in their discussion that not so much birth order but rather the length of time between births of siblings can create individual variation. Small differences in age between the child with the impairment and the sibling were proposed to lead to more negative feelings (#4) in comparison to larger age differences. In study #10, a difference was found in aspects of prosocial tendencies between older and younger siblings; younger siblings showed more public and altruistic prosocial tendencies than older siblings, but less prosocial tendencies in anonymous and compliant situations.

#### Sex

The effect of the sex of the siblings was examined in four studies. Whereas study #4 found no significant differences by sex, studies #1, 5, and 10 did. Brothers showed more externalizing behavior (#5) and a significantly higher self-concept (#1) than those of sisters, while sisters showed more prosocial behavior (#1, 5) and nurturing and dominance in sibling relationships (#5). In study #10, brothers showed more public prosocial behaviors than those of sisters, and the latter showed more emotional and altruistic prosocial behaviors.

#### Sibling behavioral and personality characteristics

According to study #5, a difficult temperament in siblings is significantly related to more externalizing and internalizing behavior. In addition, study #5 argued that a more positive temperament of siblings may help them to adapt to the situation of having a brother or sister with a disability resulting in fewer behavior problems. However, if siblings have high amounts of domestic responsibility, it may negatively impact the QoL of the sibling (#3). It was suggested that knowledge of the impairment of their sibling also may be helpful (#6, 11).

#### Parental and family characteristics

Parental and family characteristics can relate to the relationship between the child with the disability and the QoL of the sibling. Two studies took these factors into account. In study #4, siblings from bigger families reported significantly more negative feelings than siblings from smaller families. Studies #4 and 9 found that a permanent job for at least one of the parents was a protective factor for the QoL of the sibling. The discussion section of several studies implied that parental and family characteristics could be potential factors. Study #5 suggested that the stress of parents may negatively impact the QoL of siblings. In contrast, good communication between parent and child (#1, 5), parental availability (#1, 3, 5, 6, 11), and a healthy family environment (#1, 3) were suggested as positive factors for the wellbeing of siblings of children with disabilities. The culture and ethnicity of the family may also play a role in the QoL of the sibling (#5), although the specific impact was not clearly described. According to studies #6 and 10, parenting behavior may also facilitate the prosocial behavior of the siblings. Study #4 suggested that whether a child who is DHH goes to a regular school or a residential school may also have an impact on sibling relationships. The rationale is that a primarily hearing environment creates more opportunities for interaction than a residential school, which in turn stimulates sibling relationships at home.

#### Respondents' bias

QoL of siblings can differ based on who completed the report. For example, study #2 found that mothers regularly reported more problems than fathers, and study #8 found that children reported significantly fewer problems for themselves than their parents. A possible explanation for systematic differences in response is given in study #5, which mentions that parental reports can be affected by the (negative) feelings of the parents.

#### Social network

Study #1 found that classmate support was significantly and positively related to the self-concept of the siblings and the authors suggested a protective role of peer relations in sibling adjustment outcomes. Study #11 mentioned the importance of peers and participation in support groups for creating positive feelings for the sibling.

## Discussion

This scoping review examined the QoL of siblings of children who are DHH, have a VI or MI. The current review should complement previous reviews that focused on siblings with other conditions, such as chronic illnesses, intellectual disabilities, or neurodevelopmental disorders, which are not comparable to siblings of children with motor or sensory disabilities.

The first research question addressed the current state of knowledge on the QoL of siblings of children who are DHH, have a VI or MI. The included studies assessed varying developmental domains for which they used a variety of terms and operationalizations of QoL. For example, Bellin et al. (44) investigated psychological and behavioral adjustment by investigating indicators such as attitudes toward illness and self-concept. Haegele et al. (50) described psychosocial wellbeing in their introduction, which they restricted to prosocial behavior in their results section. QoL is a multifaceted construct with various operationalizations (23), which may explain the great diversity between the included studies. Because of this, we also found a large variety of assessment tools used in the studies, as many as 21 different assessment tools. It appeared that there is no generally accepted assessment tool. The problem with such a large variation in tools is that the concepts measured by these tools also differ. The variety in operationalization and the assessment tools to measure QoL has been addressed before in reviews (5, 55). Both argued that the large variety of tools and concepts reduces the possibility to draw definite conclusions or generalize results. The current review encountered the same problems.

Despite the abovementioned methodological issues, some conclusions can be drawn regarding the first research question. For clarity, the findings of the studies were categorized into themes by the first and second authors: QoL (overall), wellbeing, relationships, adjustment, mature behavior, and self-perception. These themes correspond with three of the eight domains of Schalock et al. (23), that is, personal development, interpersonal relations, and emotional wellbeing. The remaining five domains of Schalock et al. (23) were not studied in the studies. The results seemed conflicting. For example, for the theme adjustment, Laufersweiler et al. (46) found that siblings of children with MI had significantly higher scores on problem behavior compared to controls. In contrast, Raghuraman (48) found no differences in problem behaviors between siblings of children who are DHH and siblings of children with typical hearing. Of course, it is impossible to reach a decisive conclusion based on only these two results, but, at least, these results require further study with more groups.

For the first research question, we further investigated which assessment tools were used, which control group was used, and what the positive and negative consequences were for the siblings. A large variety of assessment tools were used. If the same assessment tool was used in different studies, the results seemed consistent between the studies. For the SPQ, CBCL, and SDQ, matching results were found. The use of the SDQ in three studies showed that siblings acted more prosocially than controls (45, 50), and scores were rather high compared to standard mean scores (47).

Other researchers also found positive and negative consequences for the QoL of siblings in the current review on similar topics with other groups (32, 56, 57). Small sample sizes, variations in assessment tools, and different mediating factors were identified as possible causes for inconsistency in findings (32, 56, 57), which also applies to the current review.

The second research question concerned the factors that could be associated with individual variation in QoL of siblings of children who are DHH, have a VI or MI. While studies identified several factors affecting the QoL of siblings, these have not yet been studied thoroughly. Amongst the better-studied factors are age and sex. Older siblings showed less interpersonal concerns, fear, and conflict than younger siblings (48). An explanation could be that with age the sibling without the disability acquires more self-regulation skills and therefore understand better the child with the disability and subsequent parental actions (58). Regarding sex differences, brothers showed more externalizing behavior and scored higher on self-concept than sisters, while sisters showed more prosocial behavior and nurturing and dominance in the sibling relationship. Sisters also showed more emotional and altruistic prosocial tendencies, while brothers had more public prosocial tendencies (44, 48, 53). Morawska (59) suggested that such differences may result from typical gender-specific behaviors promoted by parents leading to differences in socialization of boys and girls, which in itself is not different from siblings who do not have a brother or sister with a disability.

Next to factors within the individual, external factors impact the QoL of siblings, such as good communication within the family and parental availability (44, 46, 48, 49, 54). This variety of factors affecting the QoL of siblings is also found in previous reviews with other groups of siblings (5, 33, 60, 61). For example, Luitwieler et al. (33) extracted 98 different variables from 40 studies on the QoL of family members of children with intellectual disabilities.

The conceptualization of QoL as done by Schalock et al. (23) does not include mediating or moderating factors, in contrast to the International Classification of Functioning, Disability and Health (ICF) model (19) and the Family of Participation-Related Constructs (fPRC) framework of Imms et al. (35). This might be regarded a drawback of the QoL concept. The fPRC framework states that the experiences of an individual with a disability are determined both by factors within (intrinsic) and outside (extrinsic) the individual. Siblings are part of the context, but from the point of view of these siblings themselves, the child with the disability is a contextual factor. As such, there are mutual relations between children within a family.

### Limitations

Some limitations of this review must be considered. First, while it was known that this specific topic is relatively little researched, we restricted potential findings to peer-reviewed English scientific articles. As a result, no gray literature, for example, internal reports, were included in the review. In addition, only articles published in the last 20 years were considered, potentially excluding older but relevant studies. This choice was, however, deliberately made because more recent societal developments formed a contraindication to include older publications. Examples of such developments are as follows: improvements in technology and neonatal care; changes in rehabilitation practices by initiatives such as the Convention on the Rights of Persons with Disabilities (CRPD) and the International Classification of Functioning, Disability, and Health for Children and Youth (ICF-CY) (37, 38); and deinstitutionalization of children with disabilities in the Western world (62).

Second, the operationalization of QoL is tenuous. Roberts (5) stated that the experiences, outcomes, and needs of each sibling are unique and complex, making it difficult for research tools to fully understand individual circumstances. The studies included in the review used different assessment tools to measure QoL, and when the same tool was used, it could still measure different constructs. For instance, the SDQ was used to investigate psychological wellbeing by Hamblion et al. (51), psychological adjustment by Cianfaglione et al. (45), and behavioral adjustment by Bellin et al. (44) and Hadjikakou et al. (47). In addition, the current scoping review specifically focused on QoL of young siblings until the age of 18 years and did not include personal characteristics of the siblings in the study. It might well be that personality traits, cognitive level, and specific roles of the siblings, such as taking on the carer role affect the QoL of these siblings. For instance, it is repeatedly shown that a lot of siblings, whether or not over 18 years old, take on caring responsibilities (9, 63). Therefore, there is much more to explore about siblings of children with disabilities.

Finally, although not a limitation in the sense that the inclusion criteria were too strict, the included studies were only conducted in Europe and the United States of America. This may give a one-sided perspective, as parenting and family relations differ across countries and cultures. For example, in Asian and African countries, it is more typical to live in communities and strive for group instead of individual wellbeing compared to Western countries. As a result, demands on children and the affection shown to them differ as well (64).

### Implications

In the current scoping review, we found that many studies suggested that parenting-related variables may impact the QoL of siblings. For instance, parental availability and good communication between the family members were often suggested (44, 46, 48, 49, 54). However, such variables have hardly been studied empirically. From research on siblings of children with other disabilities or illnesses, we already know the importance of good communication between family members (65, 66). Future research should focus on the specific group of siblings of children who are DHH, have a VI or MI and investigate how communication between family members can enhance the QoL of siblings.

The implication of focusing on parenting-relating variables fits perfectly with current trends in rehabilitation to focus on family-centered care (67). The information helps healthcare providers apply more effective rehabilitation (56). In some countries, an integrative approach is already included in rehabilitation. For example, in the United States of America, Individual Family Services Plans (IFSP) are mandatory for families with a child with a disability (68). The IFSP addresses not only the needs of the child but also the other family members (69). We suggest other countries to adopt this plan to ensure QoL for everyone.

Siblings often act as young carers (9, 63). Care responsibilities and less time can impact their participation in leisure activities. Therefore, a research suggestion is to use, next to QoL, participation as an outcome. As participation is likewise a multifaceted concept, a framework that describes participation, such as fPRC (35), can guide future research and operationalize outcomes. With the fPRC framework, Imms et al. (35) proposed that the experiences of an individual with a disability are determined not only by intrinsic factors but also by extrinsic factors in their context and environment. Whereas intrinsic factors, such as self-esteem, are placed within the individual, extrinsic factors refer to the context (e.g., parenting skills or home environment) and the environment (neighborhood or societal facilities).

Within interventions, a distinction is made between modifiable and non-modifiable factors (56). For example, age is non-modifiable. Interventions should focus on modifiable factors such as the parent–child relationship. This can be complemented by using the fPRC framework. To help clinicians, a simple assessment technique is to divide intrinsic/extrinsic from modifiable/non-modifiable factors (35, 56) in a schema with four quadrants. For example, parenting characteristics are an extrinsic and modifiable factor, and behavior characteristics are intrinsic and modifiable. Birth order and impairment of the child are examples of extrinsic and non-modifiable factors. Finally, age and sex are intrinsic and non-modifiable. Taking these different factors into account, the strengths and difficulties of siblings of children with disabilities can be better understood and assessed, and the subsequent intervention aimed at modifiable factors. In the current review, we found different behaviors for brothers or sisters of children with disabilities (44, 48, 53). Sex is an intrinsic, non-modifiable factor. This could mean that healthcare providers prepare different interventions for brothers and sisters of children with disabilities. The included studies also suggested that parental availability can be helpful for the QoL of siblings (44, 46, 48, 49, 54). This is an extrinsic, modifiable factor. Healthcare providers can train and support parents in how to interact with their child without disability and enhance their parental availability.

To our surprise variables such as socioeconomic status, caregiver education and access to resources were not mentioned to account for individual variation in QoL of siblings. Given their universal importance for early intervention (11), we would like to suggest studying these factors in future studies. Although such variables are not modifiable, awareness of these variables by healthcare providers is important. Information about the role of the non-modifiable variables helps recognize which siblings need extra attention because of an increased risk of a reduced QoL (56).

Future research on the QoL of siblings should consider whose information is used. Half of the studies analyzed the QoL of siblings with information from parents, not of the siblings themselves, which carries the risk of a one-sided focus (33). The emotions of parents can influence their assessment of the QoL of children (48). For example, Hamblion et al. (51) found that parents reported more behavioral problems for the siblings than the siblings did themselves. To gain a sound understanding of the QoL of siblings of children who are DHH, have a VI or MI and important factors for individual variation, it is therefore recommended to include the perspective of the siblings themselves.

## Conclusion

The present scoping review provides an overview of the current knowledge on the QoL of siblings of children who are DHH, have a VI or MI, based on research from 2003 to 2021. Limited research specifically focused on the QoL of siblings of children who are DHH, have a VI or MI. The review included 11 studies that examined the QoL of siblings, using tools such as the SDQ and CBCL. Negative consequences observed in the QoL of the siblings include higher scores on depression (52) and internalizing behavior (46) compared to siblings of children without disabilities. Positive consequences include better psychological adjustment compared to norms (45) and better adjustment in siblings who perceived family satisfaction (44). Various factors relate to the QoL of siblings, suggesting opportunities for future research with an integrative approach and interventions particularly focusing on modifiable variables such as family communication and parenting behavior.
