# Supplementary material for: Scoping review: quality of life of siblings of children who are deaf and hard of hearing, have a vision or motor impairment
Source: Front Rehabil Sci. 2023 Nov 14;4:1227698. doi: 10.3389/fresc.2023.1227698 (PMC10682732; doi:10.3389/fresc.2023.1227698)
Supplement: Supplementary file 1 [file Table1.pdf]

Supplementary file 1. Searchstring PsychInfo

1	Sibling*.mp
2	“wellbeing” OR “well-being” OR (well ADJ2 being).mp
3	“psychological adjustment”.mp
4	(quality ADJ2 life).mp
5	(life ADJ2 satisfaction).mp
6	(problem ADJ2 behavio*).mp
7	Disabilit*.mp
8	Disease*mp
9	Disorder*.mp
10	Syndrome*.mp
11	Impairment*.mp
12	2 OR 3 OR 4 OR 5 OR 6
13	7 OR 8 OR 9 OR 10 OR 11
14	1 AND 12 AND 13
15	Limit 14 to yr=”2002-current”
