# Supplementary material for: Scoping review: quality of life of siblings of children who are deaf and hard of hearing, have a vision or motor impairment
Source: Front Rehabil Sci. 2023 Nov 14;4:1227698. doi: 10.3389/fresc.2023.1227698 (PMC10682732; doi:10.3389/fresc.2023.1227698)
Supplement: Supplementary file 2 [file Table2.pdf]

Supplementary file 2. Selection inclusion form

Authors:

Title:

DOI:

1. Are siblings of children who are deaf and hard of hearing, have a vision impairment or motor impairment object of the study?

   Yes ☐ Go to question 2 No ☐ Reject

2. Is the initial age of the participants between zero and 18 years?

   Yes ☐ Go to question 3 No ☐ Reject

3. Does it apply siblings of children who are deaf and hard of hearing, have vision impairment or motor impairment?

   Yes ☐ Reject No ☐ Go to question 4

4. Are outcomes related to QoL of siblings of children who are deaf and hard of hearing, have vision impairment or motor impairment?

   Yes ☐ Go to question 5 No ☐ Reject

5. Does it involve a study with empirical data?

   Yes ☐ Retain the paper. No ☐ Reject

6. Is the study written in English and peer-reviewed?

   Yes ☐ Retain the paper. No ☐ Reject
