# Supplementary material for: Scoping review: quality of life of siblings of children who are deaf and hard of hearing, have a vision or motor impairment
Source: Front Rehabil Sci. 2023 Nov 14;4:1227698. doi: 10.3389/fresc.2023.1227698 (PMC10682732; doi:10.3389/fresc.2023.1227698)
Supplement: Supplementary file 3 [file Table3.pdf]

Supplementary file 3. Data extraction form

General information	
	Author
	Year
	Country of origin
	DOI:
Study design	
	Setting
	Method
Sample	
	Sample size
	Composition sample
Participants	
	Average age
	Type of impairment
Assessment tool	
	Domain
	Assessment tool
Outcomes	
	Outcomes reported in results
	Possible explanations in conclusion and discussion
